# Podcasting in primary care: attitudes of Scottish GP specialty trainees and trainers towards podcast-based education in primary care

**DOI:** 10.3399/BJGPO.2024.0248

**Published:** 2025-12-19

**Authors:** Varun Rana, Blair H. Smith, Callum J Leese

**Affiliations:** 1 University of Dundee Medical School, Ninewells Hospital, Dundee, UK; 2 Department of Population Health and Genomics, University of Dundee, Ninewells Hospital, Dundee, UK

**Keywords:** education, general practice, podcast, training

## Abstract

**Background:**

Podcasts are rapidly gaining popularity within medical education, but their acceptability and effectiveness within primary care education remains understudied.

**Aim:**

To evaluate the attitudes of general practice specialty trainees (GPSTs) and GP trainers towards audio podcasts for primary care medical education.

**Design & setting:**

A cross-sectional questionnaire distributed to all GPSTs and GP trainers in Scotland.

**Method:**

The survey evaluated podcast usage patterns, their perceived effectiveness, and perspectives on the benefits and challenges of podcast-based education. Quantitative data were analysed using descriptive and inferential statistics, and qualitative data underwent thematic analysis.

**Results:**

Of 1995 invited participants, 219 individuals (11.0%) responded. Medical education podcasts were used by 69.9% of responders, with higher usage among GPSTs (73.5%) than GP trainers (65.7%). Most responders (89.0%) perceived podcasts as an effective medium for primary care education. The majority of responders noted improvements in professional knowledge (92.8%), confidence in delivering patient care (73.5%), and clinical care provision (81.6%), as a result of podcast usage. Key benefits of podcasts included flexibility and accessibility, while the main challenge was personal time constraints. Responders highlighted quality assurance and awareness as key areas for improving the experience of podcast-based education.

**Conclusion:**

Podcasts are accepted and perceived as an effective educational tool within primary care education. Future efforts should focus on developing high-quality, relevant podcasts, and addressing concerns around personal time constraints and quality assurance. Further research, including larger, more representative samples, is needed to assess the impact of podcasts on knowledge retention, clinician behaviour, and clinical outcomes within primary care.

## How this fits in

Podcasts are becoming increasingly popular within medical education, but their use within primary care education remains largely unexplored. Previous studies in other medical specialties have shown podcasts to be a valuable educational tool, but research specific to primary care has been limited. This study provides the first comprehensive assessment of the acceptability and perceived effectiveness of podcasts among primary care doctors, both globally and in Scotland. Our findings demonstrate podcasts to be widely accepted and perceived as an effective educational medium, highlighting their potential as a valuable tool within primary care education.

## Introduction

Podcasts have seen an exponential growth in production and popularity across the world.^1^ In 2021, a Google search on ‘podcast’ produced over 1.9 billion search results, in comparison with only 6000 search results in 2004.^2^ Within the UK, the estimated number of podcast listeners between 2017 and 2020 has increased from 8.99 million to 15.61 million.^1^

In parallel to the growth of podcast popularity within society, there has been a similar trend within medical education, with podcasts being used by a variety of healthcare professionals.^3^ Podcasts are also available across all medical specialties, particularly within emergency medicine, which has led the way in the adoption of this learning medium.^4^

The growth in the popularity of podcasts within healthcare education can be attributed to several key factors including their ability to quickly distribute current information, to provide listeners with diverse viewpoints, and to offer on-demand learning for busy healthcare professionals, of varying levels of expertise.^5–7^ In addition to these factors, the COVID-19 pandemic significantly impacted the delivery of medical education, accelerating the adoption of remote learning methods, including podcasts.^8^

Despite the large number of medical podcasts available, the evidence base supporting their value as an education tool remains limited. A recent scoping review of podcasting within medical education^9^ found that learners valued podcasts for their flexibility, engagement, and efficiency. The scoping review highlighted positive changes in learner behaviour and practice from podcast-based education; however, no studies evaluated the resulting impact on clinical and patient outcomes, and this remains a key area for research.

While podcasts have been increasingly recognised as a valuable educational resource, primary care professionals have been slower to adopt this medium compared with their counterparts in other medical specialties.^10^ However, there is now a wide range of podcasts specifically tailored to primary care, covering a broad spectrum of clinical and lifestyle-related subjects. The research into podcasting within primary care education is sparse, with only two relevant publications in this area. A conference abstract by Brust and colleagues^11^ found podcasts to be comparable with traditional lectures, in terms of efficacy and learner satisfaction. The second publication reported an evaluation of educational needs for primary care allergy teaching, finding podcasts to be a less favourable educational medium in comparison with others such as online guidelines and courses.^12^

Consequently, this study aims to evaluate the attitudes of general practice specialty trainees (GPSTs) and GP trainers towards audio podcasts for primary care medical education.

## Method

### Study design

A cross-sectional survey with qualitative and quantitative aspects was developed (using an online platform; Jisc Online Surveys [JOS] tool) to evaluate the acceptability and perceived effectiveness of podcasts within primary care education. The questions were compiled by an advisory panel, including medical education specialists (*n* = 2), academic researchers (*n* = 2), a GP trainer (*n* = 1), and a GPST (*n* = 1). The survey (Supplementary Appendix 1) included 12 questions, combining binary (yes or no), five-point Likert scale, and free-text questions. The study design was informed by a previous study by Leese and colleagues.^13^ Before distribution, the survey was piloted (by GPSTs and GP trainers) to ensure its functionality and content validity.

### Participation selection

This study was conducted in collaboration with NHS Education for Scotland (NES), which is the administrative body responsible for all GPSTs and GP trainers in Scotland. GPSTs represent the future workforce in primary care and GP trainers have valuable insights into primary care education. These groups were involved in the study to gain a varied understanding of podcast use within primary care education.

### Data collection

The survey was distributed, via email, to all GPSTs and GP trainers in Scotland by NES on 22 February 2024. Reminder emails were sent on 7 March 2024 and 19 March 2024, and the survey closed on 21 March 2024.

### Data analysis

Data were first downloaded and cleaned in Microsoft Excel before analysis. Descriptive statistics were used to present the quantitative data. Data were imported into GraphPad Prism (version 10.3.1) and inferential statistics were applied using the Fisher’s exact test to compare responses between GPSTs and GP trainers. Survey responses for the open-ended qualitative questions were analysed using a conventional approach to content analysis, as outlined by Hsieh and Shannon(^14^).^15^Free-text responses were analysed within Microsoft Excel. The analysis process for each open-ended question involved the following five stages: (a) authors (VR and CL) thoroughly reviewed all free-text responses to become familiar with the data; (b) each response was analysed and coded based on emerging sub-themes; (c) these sub-themes were then grouped into broader themes based on similarities and differences; (d) themes were further refined in an iterative process to ensure all relevant themes were captured; (e) a frequency analysis was conducted to assess the frequency of certain themes within the data. The analysis was primarily performed by one author (VR), with key stages discussed and validated by a co-author (CL) to ensure reliability.

## Results

### Demographics

Of the 1995 individuals (1205 GPSTs and 750 GP trainers) invited to participate in this study, 219 (11.0%) responded to the survey.

Of responders, 117 (53.4%) were GPSTs. This represented 9.7% of all GPSTs in Scotland (*n* = 117/1205). Of the 117 GPSTs who responded, 34.2% (*n* = 40) were in their first year of training (GPST1), 25.6% (*n* = 30) were in their second year (GPST2), and 39.3% (*n* = 46) were in their final year (GPST3). One GPST (0.9%) was ‘Out of Programme’.

Of all GP trainers in Scotland (*n* = 750), 13.6% (*n* = 102) responded to the survey, forming 46.6% of the study sample.

### Acceptability and use

One hundred and fifty-three responders (69.9%) reported listening to medical education podcasts. This represented 73.5% (*n* = 86) of GPSTs and 65.7% (*n* = 67) of GP trainers.

**Figure 1.**
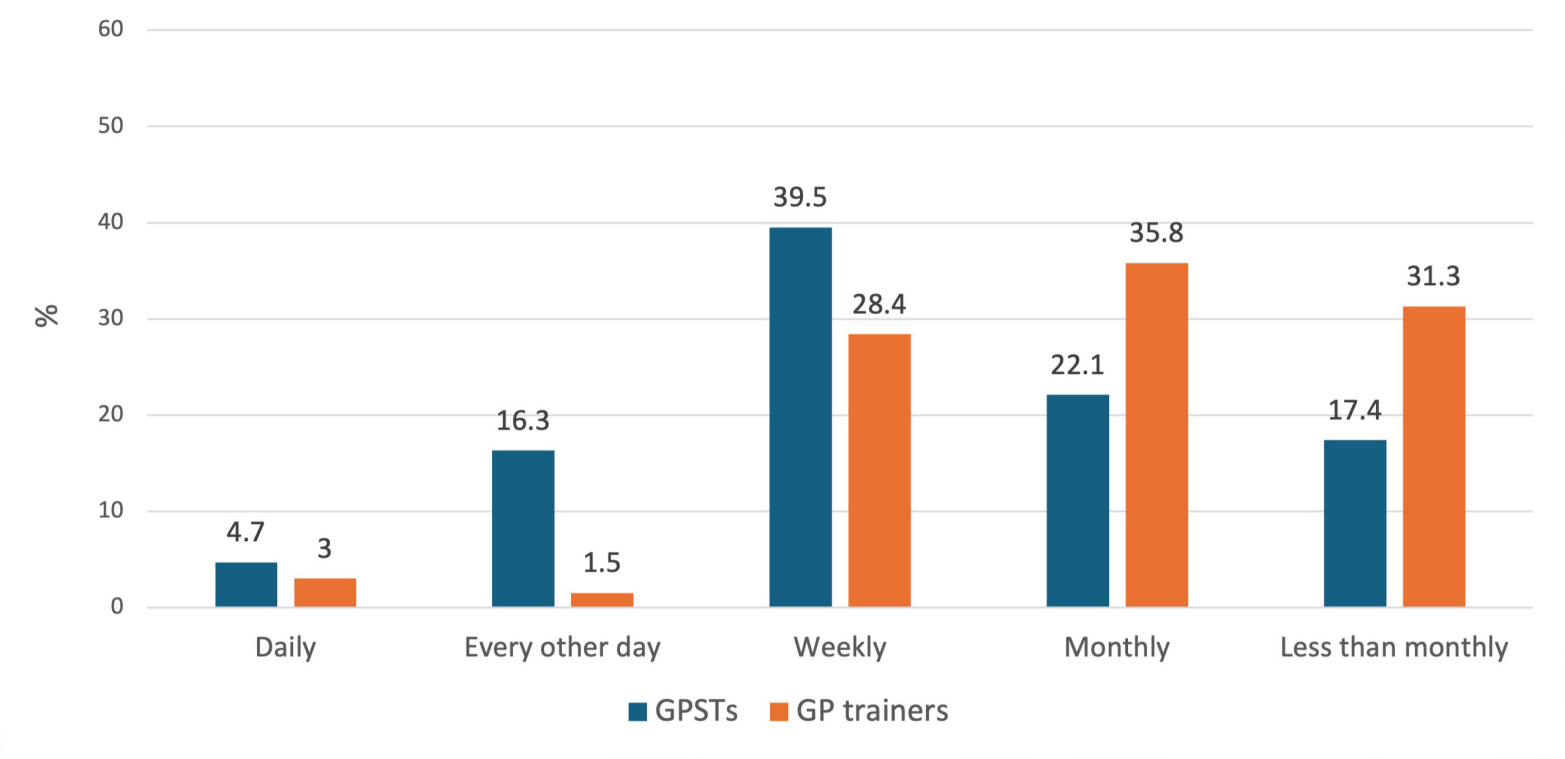
Frequency of medical education podcast listening among GPSTs and GP trainers. GPST = GP specialty trainee

Responding GPSTs reported listening to medical education podcasts significantly more frequently than GP trainers (*P* = 0.002). GPSTs mostly listened on a weekly basis (39.5%, *n* = 34). In comparison, GP trainers mostly listened on a monthly basis (35.8%, *n* = 24) (Figure 1).

The majority of responding GPSTs (86.0%, *n* = 74) and GP trainers (98.5%, *n* = 66) reported using a single medium for podcast listening. Smartphones emerged as the most popular medium for both GPSTs (97.7%, *n* = 84) and GP trainers (95.5%, *n* = 64). Computers or laptops were used by 8.1% (*n* = 7) of responding GPSTs and 1.5% (*n* = 1) of GP trainers.

Most responding GPSTs (89.5%, *n* = 77) and GP trainers (94.0%, *n* = 63) listened to medical education podcasts in their own time.

The majority of responders listened to podcasts when travelling, representing 91.9% (*n* = 79) of responding GPSTs and 71.6% (*n* = 48) of GP trainers. Other popular locations included at home (55.8% of GPSTs and 50.7% of GP trainers) and outdoors (34.9% of GPSTs and 47.8% of GP trainers). No GPSTs, and only one (1.5%) GP trainer listened to medical education podcasts at work.

### Perceived effectiveness

The majority of responding GPSTs (91.5%, *n* = 107/117) and GP trainers (86.3%, *n* = 88/102) believed podcasts to be an effective way of delivering primary care education (89.0% of total responders).

On the whole, responding GPSTs (96.5%, *n* = 83/86) and GP trainers (88.1%, *n* = 59/67) felt that listening to podcasts had improved their professional knowledge (92.8% of total responders). GPSTs were significantly more likely to report that podcasts improved professional knowledge compared with GP trainers (*P* = 0.046, standard error [SE] 0.04). A single GP trainer (and no GPSTs) reported that podcasts had not improved their professional knowledge.

Most responding GPSTs (77.6%, *n* = 66/85) and GP trainers (68.2%, *n* = 45/66) believed they were more confident in delivering patient care as a result of listening to podcasts. Only one GPST and one GP trainer reported that podcasts did not increase their confidence in delivering patient care.

**Figure 2.**
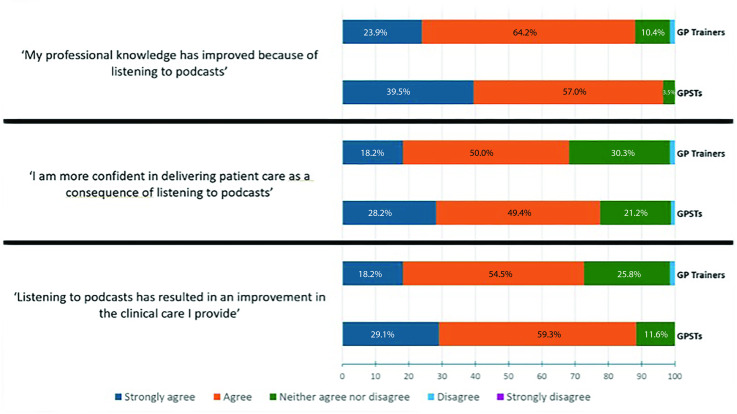
GPSTs’ and GP trainers’ responses to statements regarding medical education podcasts. GPST = GP specialty trainee

The majority of responding GPSTs (88.4%, *n* = 76/86) and GP trainers (72.7%, *n* = 48/66) felt listening to podcasts had resulted in an improvement in the clinical care they provide. This represents 81.6% (n=124/152) of total respondents. GPSTs were significantly more likely to report that podcasts improved their clinical care compared with GP trainers (*P* = 0.043, SE 0.04). No GPSTs and one GP trainer indicated that using podcasts did not improve the clinical care they provide (Figure 2).

### Thematic analysis

#### Benefits of podcasts

The following three themes emerged from the free-text responses: flexibility and accessibility (78.9%, *n* = 105/133); diversity of offering (35.3%, *n* = 47/133); and autonomy of learning (9.0%, *n* = 12/133). Table 1 provides a summary of these themes, including illustrative quotes.

**Table 1.** Themes relating to the benefits of podcasts for primary care education

Theme	Theme description	Percentage of total responders (*n* = 133)	Illustrative quotes
Flexibility and accessibility	Podcasts are convenient and accessible, and can be listened to while doing other activities, such as commuting or exercising	78.9%	*'Flexibility in engaging with teaching, ability to you revise in natural gaps in the day (commute) or while doing other tasks (chores)* […]' GPST *'easily accessible on the go, can multitask and revise/ lear*[n] *simultaneously.' GPT* *'The opportunity for up-to-date information to be disseminated / expert opinion to be shared in a brief format is invaluable.' GPST*
Diversity of offering	Podcasts cover a variety of topics, accommodate different time restraints, support auditory learners and offer an engaging alternative to reading	35.3%	*'For people with neurodiversity or dyslexia podcasts can be particularly helpful.* […] *Listening is much easier and more appealing to me than reading.' GPT* *'I have listened to bitesize podcasts for studying for my AKT and I found these helpful consolidating my learning from reading/practise questions prior to the exam.' GPST* *'Short podcasts if you have a 10-minute drive to a house call might be great or an hour looking at something more in depth if you are at home.' GPT*
Autonomy of learning	Podcasts can be paused and replayed to enhance learning, and can be used offline	9.0%	*'I can play back areas I didn't catch or understand.' GPST* *'Can be listened to in chunks/start and stop as you want.' GPST* *'Doctor directed learning. Own time to reflect.' GPT*

AKT = Applied Knowledge Test. GPST = GP specialist trainee. GPT= GP trainer.

#### Challenges of podcasts

The following five main challenges were described by responders: time constraints (36.5%, *n* = 50/137); lack of perceived retention (25.5%, *n* = 35/137); podcast credibility (16.8%, *n* = 23/137); accessibility and convenience issues (13.9%, *n* = 19/137); and lack of relevance (5.1%, *n* = 7/137). Table 2 provides a summary of these themes and several illustrative quotes.

**Table 2.** Themes relating to the challenges of podcasts for primary care education

Theme	Theme description	Percentage of total responders (*n* = 137)	Illustrative quotes
Time constraints	Difficult to find time within busy schedule to listen to podcasts, and a preference for non-medical content during personal time	36.5%	*'Finding the time to listen is still a challenge since this happens mostly during personal time.' GPST* *'Tired at end of day, want to switch off and listen to something else rather than medicine in the car.' GPT* *'Length, sometimes too long.' GPST*
Lack of perceived retention	Poor knowledge retention owing to mismatched learning styles, lack of interactivity, and distractibility while listening	25.5%	*'Some people learn better by visual/face to face. They are generally pre-recorded so no way to clarify/ask questions.' GPT* *'Not sure how actively engaged you are as risk of passive listening.' GPT* *'May be hard to concentrate if stressed or distracted.' GPT* *'I find my recall of information is not as good from a podcast as it is from a presentation or from reading* […]' GPST
Podcast credibility	Difficulty in identifying trustworthy podcasts amid the growing number of available sources, and uncertainty surrounding the evidence base of podcasts	16.8%	*'Can be uncertain of source validity — when is a podcast fact versus opinion?' GPT* *'Having a trustworthy site and a trustworthy podcast. What is the podcaster’s agenda?' GPT* *'Risk of information not being evidence based — more difficult to fact check information if not reading a paper with references.' GPST*
Accessibility and convenience issues	Unfamiliarity with podcast platforms, difficulty taking notes while multitasking, and challenges in navigating podcasts compared with text-based resources	13.9%	*'If we have hearing problems, concentration problems or no access to spotify or headphones then I can imagine this would reduce accessibility.' GPT* *'In addition it is much harder to skip irrelevant information or to locate particular information.'* *'Difficult to take notes or "prove" learning.' GPT*
Lack of relevance	Difficult to find podcasts relevant to specific educational needs, noting a limited variety of primary care podcasts and lack of UK or Scotland-specific content	5.1%	*'Sometimes the ones available aren't relevant to my location.' GPST* *'Very few Scottish-based podcasts for GPs and GP trainees.' GPST* *'A few of the more popular podcast I have found are American which are not as useful as they have different managements / different units when talking about investigations* […]*' GPST*

GPT = GP Trainer; GPST = GP specialist trainee;

#### Improving the experience of podcasts

The following four themes emerged from the suggestions of responders: content and format (34.7%, *n* = 33/95); awareness (28.4%, *n* = 27/95); relevance (18.9%, *n* = 18/95); and quality assurance (13.7%, *n* = 13/95). Table 3 provides an overview of these themes and illustrative quotes from responders.

**Table 3.** Themes relating to improving the experience of podcasts for primary care education

Theme	Theme description	Percentage of total responders (*n* = 95)	Illustrative quote(s)
Content and format	Importance of engaging and practical podcast content, suggesting a mix of clinical and non-clinical topics, concise podcasts with summaries, interactive elements such as quizzes, and easy, free access through popular platforms	34.7%	*'Good practical topics, also presented in a concise way, not too long, interactive and practical.' GPST* *'Reliable source, clarity in audio, relatable topics, able to access offline.' GPST* *'Maybe a quiz you can access after listening to the podcast.' GPT*
Awareness	Improving awareness of high-quality medical education podcasts, through the provision of curated lists by trusted sources such as NES or RCGP, and sharing recommendations through newsletters or conferences	28.4%	*'NES to provide list of useful podcasts for GPs / GP trainees.' GPST* *'Better signposting to good resource (currently really just word of mouth).' GPT* *'Easy list or source of reliable good-quality medical education podcasts.' GPT*
Relevance	Need for UK and Scotland-specific podcasts, addressing local guidelines and challenges. GPSTs requested podcasts that help prepare them for GP examinations	18.9%	*'Scottish/UK base guideline approach to primary care would be useful.' GPST* *'Take into account challenges of living in parts of Scotland where some tests/treatments etc may not be available.' GPST* *'Tailoring the podcasts to the needs of a GPST preparing for RCGP examinations.' GPST*
Quality assurance	Need for podcasts to provide accurate, reliable, and trustworthy information, suggesting a peer-review process or external evaluation by recognised institutions such as NES or RCGP	13.7%	*'Validity testing/endosrsement by NES for example.' GPST* *'Some external evaluation of quality and safety of the content — a certification process.' GPT* *'I don't know whether there is e.g., some sort of peer-reviewing process for some podcasts — I would probably trust them a little more.' GPST*

GPST = GP specialty trainee. GPT = GP trainer.﻿ NES = NHS Education for Scotland. RCGP = Royal College of General Practitioners.

## Discussion

### Summary

Notwithstanding the relatively low response rate (11.0%), we found that more than two-thirds (69.9%) of GPSTs and GP trainers who responded to our survey reported listening to medical educational podcasts, suggesting they are generally an acceptable medium for delivering primary care education.

Furthermore, podcasts are widely perceived as an effective way of delivering medical education, with 89.0% of GPSTs and GP trainers sharing this belief. GPSTs and GP trainers believed that podcasts had improved their professional knowledge, increased their confidence in delivering patient care, and enhanced the clinical care they provide.

The thematic analysis highlights the flexibility and accessibility of podcasts as one of their key benefits. Despite this, a number of challenges exist, including concerns regarding podcast credibility, personal time constraints, and poor knowledge of resource availability.

### Strengths and limitations

This is the first study (to the best of our knowledge), globally or in Scotland, to assess the acceptability and perceived effectiveness of podcasts in primary care education. The analysis of this study was performed on a small number of responders (*n* = 219), that represented 11.0% of all GPSTs and GP trainers in Scotland. Because of this, care should be taken to not overgeneralise the results of this study. Although the response rate compares with recent, similar studies,^13,16^ it may have been influenced by pressures on primary care at the time of the survey owing to health economic circumstances and the relatively short-time window for which the survey was open. Given the response rate and these pressures, responder bias may have been amplified in these results, including more responders who have a pre-existing interest in medical education podcasts. Future work should consider the utilisation of incentives for participation,^17^ and the adoption of a more extensive communication strategy to maximise survey response rate. Thematic analysis of the free-text responses offered valuable insights into the perceptions of GPSTs and GP trainers, but the absence of in-depth methods, such as focus groups or semi-structured interviews, limited the depth of the qualitative data, and should be considered in future studies.

### Comparison with existing literature

The high prevalence of podcast use among GPSTs and GP trainers, particularly while multitasking, corroborates findings from other studies into podcast use across other specialties.^5,7,18^ Our study extends these findings to the primary care context, further emphasised by responders identifying the flexibility and accessibility of podcasts as a key benefit for time-constrained GPSTs and GP trainers.

The more frequent use of podcasts and greater likelihood of reporting improvements in professional knowledge and clinical care reported by GPSTs compared with GP trainers may be influenced by several factors including differences in the following: (a) life circumstances; (b) existing professional knowledge; (c) the content of podcasts and the audience to which they are aimed; and (d) generational technology adoption. Although our study did not specifically collect data on participant age, it is likely that GPSTs are younger on average, and as suggested by Purdy and colleagues,^15^ more likely to be technologically proficient, and to utilise free online educational resources. This aligns with findings from Ryan and colleagues,^12^ who observed an age gradient in the preference for e-learning modalities among GPs, with younger GPs showing greater preference compared with their older colleagues. The greater preference shown by GPSTs in our study suggests that the integration of podcasts into primary care education could be particularly beneficial for trainees.

There is growing evidence in the literature supporting the positive influence of podcasts on clinical practice, across various specialties.^19,20^ While our study showed high levels of self-reported improvements in clinical care, no published studies have examined the impact of podcasts on patient outcomes in primary care, and remains an important area for future research. The Kirkpatrick model^21^ offers structure for subsequent studies, through four defined levels of evaluation: reaction, learning, behaviour, and results.

Our findings suggest that podcasts are perceived to be an effective teaching method within primary care education. Recent research has shown that podcasts are comparable with traditional teaching methods in terms of knowledge retention. One study (*n* = 130) demonstrated significantly higher knowledge gains arising after podcasts compared with text-based learning in orthopaedics education,^11^ while another smaller study (*n* = 49) showed greater improvement in post-test scores among podcast users compared with lecture attendees in neurology education.^22^ However, these RCTs are limited by their small sample sizes and short-term knowledge assessment periods, therefore warranting future research.

Time constraints emerged as a significant challenge, with responders citing difficulties in allocating time to podcast listening. A study by Chin and colleagues^23^ reported lack of time as the primary reason for medical students not completing educational podcasts. Matava and colleagues^24^ found that anaesthesia residents favoured short podcasts ranging from 5–15 minutes; however, further research is required to determine the ideal podcast length more accurately, with some free-text responses in our study indicating a preference for shorter podcasts.

Responders reported a lack of perceived knowledge retention from podcast-based learning, particularly when multitasking. This concern aligns with Riddell and colleagues,^25^ who noted emergency medicine residents perceived podcast learning as passive, potentially leading to reduced retention and clinical application. However, recent studies by Gottlieb and colleagues^26,27^ challenge this perception, as they found that knowledge acquisition and retention from podcasts was not significantly impaired by concurrent activities. These contrasting findings suggest a potential disconnect between perceived and actual knowledge retention, warranting further research in this area. To address these concerns, incorporating active learning strategies into podcast design, such as integrated questions, as evaluated by Weinstock and colleagues,^28^ could enhance engagement and knowledge retention.

Quality assurance emerged as another major concern, with responders expressing uncertainty about the reliability and evidence base of podcast content. This mirrors previous concerns highlighted by emergency medicine trainees.^29^ Implementing a quality assurance framework, as proposed by Paterson and colleagues,^30^ could help address these concerns regarding primary care podcasts.

### Implications for research and practice

Educational institutions, such as NES and the Royal College of General Practitioners (RCGP), could look to allocate resources to the development of high-quality primary care podcasts. These podcasts could be created in the context of national and local guidelines and topics, in order to be more relevant for podcast users. Podcasts could also be promoted through newsletters, websites, and educational conferences to increase their exposure within medical education.

A quality-assurance process could be implemented, to address concerns over podcast content accuracy and reliability, through peer-review processes or guidelines for content creation. In addition to this, educational organisations could curate lists of high-quality, relevant podcasts for GPs.

This research supports the need for future randomised controlled trials (RCTs) that evaluate the impact of medical education podcasts on knowledge retention, clinical care provision, and patient outcomes within primary care. Patient and public involvement should be considered, to ensure clinical relevance of podcasts, and to guide assessment of their clinical impact.

In conclusion, this study suggests that podcasts are accepted and perceived as an effective educational tool within primary care education. Future efforts should focus on developing high-quality, relevant podcasts, and addressing concerns around personal time constraints and quality assurance. Further research, including larger, more representative samples, is needed to assess the impact of podcasts on knowledge retention, clinician behaviour, and clinical outcomes within primary care.

## Data

The dataset relied on in this article is available from the corresponding author on reasonable request.
